# A nationwide pilot study on breast cancer screening in Peru

**DOI:** 10.3332/ecancer.2023.1494

**Published:** 2023-01-09

**Authors:** Jhajaira M Araujo, Andrea C Gómez, Winston Zingg-De Jongh, Jhon Ausejo, Iván Córdova, Luis J Schwarz, Denisse Bretel, Williams Fajardo, Luis G Saravia-Huarca, Joshuan Barboza-Meca, Zaida Morante, Juan R Guillén, Henry Gómez, Nadezhda K Cárdenas, Lady Hernández, Walter Melo, Cynthia Villarreal-Garza, Christian Caglevic, Carolina Palacio, Héctor García, Gerson Mejía, Claudio Flores, Carlos Vallejos, Joseph A Pinto

**Affiliations:** 1 Centro de Investigación Básica y Traslacional, AUNA-Ideas, Guardia Civil 571, San Borja, Lima 16036, Peru; 2 Escuela Profesional de Medicina Humana, Universidad Privada San Juan Bautista, Chorrillos, Lima 15067, Peru; 3 Department of Molecular and Cellular Medicine, Texas A&M University, TX 77843, E.E.U.U., USA; 4 Escuela Profesional de Medicina Humana-Filial Ica, Universidad Privada San Juan Bautista, Ica 11004, Peru; 5 Grupo de Estudios Clínicos Oncológicos Peruano (GECOPERU), Santiago de Surco, Lima 15038, Peru; 6 Servicio de Medicina Interna, Hospital Nacional Dos de Mayo, Lima 15003, Peru; 7 Servicio de Medicina Interna, Hospital Regional de Ica, Ica 107, Peru; 8 Universidad Norbert Wiener, Lima 15046, Peru; 9 Hospital Félix Torrealva Gutiérrez- ESSALUD, Ica 11001, Peru; 10 Centro de Cáncer de Mama, Hospital Zambrano Hellion, Tecnológico de Monterrey, 66278 San Pedro Garza García, Monterrey, México; 11 Cancer Research Department, Fundacion Arturo Lopez Perez, Santiago de Chile 7500921, Chile; 12 Departamento de Oncología Clínica, Instituto de Cancerología Las Américas - AUNA, Medellín 050022, Colombia; 13 Facultad de Medicina, Universidad de Antioquia, Medellín 050010, Colombia; 14 Departamento de Oncología Médica, Hospital Clínico VIEDMA, Cochabamba 0304, Bolivia

**Keywords:** breast cancer, health knowledge, attitudes, practice, breast neoplasms/diagnosis, breast neoplasms/prevention & control

## Abstract

**Introduction:**

A high prevalence of advanced breast cancer (BC) is a common scenario in Latin America. In Peru, the frequency of BC at Stages III/IV is ≈50% despite implementation of a programme for breast cancer screening (BCS) along the country. We carried out a study to assess the feasibility and develop an instrument to evaluate the knowledge, barriers and perception about BCS in a nationwide pilot study in Peru among candidates for BCS.

**Methods:**

We conducted a systematic review of 2,558 reports indexed in PubMed, Scopus, Web of Science, Medline-Ovid and EMBASE, regarding to our study theme. In total, 111 were selected and a 51-items survey was developed (eight items about sociodemographic characteristics). Patients were recruited in public hospitals or private clinics, in rural and urban areas of nine departments of Peru.

**Results:**

We surveyed 488 women from: Lima (150), Cajamarca (93), Ica (59), Arequipa (56), Loreto (48), Ancash (38), Junín (15), Puerto Maldonado (15) and Huancavelica (14); 27.9% of them were from rural areas. The mean of age was 53.3 years (standard deviation ± 9.1). Regarding education level, 29.8% had primary, 33.2% secondary and 37.0% higher education. In total, 28.7% of women did not know the term ‘mammogram’ and 47.1% reported never receiving a BCS (36.9% from urban and 73.5% from rural population). In women that underwent BCS, only 67% knew it is for healthy women. In total, 54.1% of patients had low levels of knowledge about risk factors for BC (i.e. 87.5% of women respond that injuries in the breast produce cancer). Cultural, economic and geographic barriers were significantly associated with having a mammogram where 56.9% of participants considered a cost ≤ 7 USD as appropriate. Mammogram was perceived as too painful for 54.9% of women. In addition, women with a self-perception of low-risk for BC and a fatalistic perception of cancer were less likely to have a BCS.

**Conclusion:**

We found that it is feasible to conduct a large-scale study in Peru. The results of this pilot study highlight an urgent need of extensive education and awareness about BCS in Peru.

## Introduction

Cancer is generally thought to be a predominant disease in developed nations. However, for breast cancer (BC), 50% of cases and 58% of deaths occur in developing nations [1]. Although the incidence is evenly split between developed and developing nations, the latter experience a much greater mortality rate. Some of the causes for the discrepancy in the mortality rate are lack of early detection, inadequate diagnosis and sub-par treatment facilities [2].

In 2013, according to the American Cancer Society, 21.77% of BC diagnosis found the cancer *in situ*. A remarkably high number, highlighting the advance screening process and public awareness in the United States. As for the diagnosis of invasive cases, the Surveillance, Epidemiology and End Results Programme reported that from 2013 to 2017, 64.71% of cases were of localised BC, 27.52% of cases were of regional cancer, 5.7% were distant cases and 2.07% of unstaged diagnosis. This data demonstrates a high amount of early diagnosis per incidence rate in the United States, aiding the low mortality rate present in the country [3].

Latin America presents a more severe situation. According to Global Cancer Observatory (GLOBOCAN) 2020, the mortality-per-incidence (MPI) ratio for BC in Peru, Chile and Ecuador’s is 0.27, 0.31 and 0.30, respectively. Although the other countries in Latin America present improved ratios, none are close to United States’ MPI (0.18) [4].

Peru has a high prevalence of advanced BC, approximately 50% of cases are at stage III/IV [1]. The primary factors associated to late diagnosis are lack of awareness, misconceptions about BC causes and treatment outcomes, social status and educational level [5, 6]. Besides, the guidelines should take into consideration the public knowledge, and social and cultural barriers in order to implement better strategies that motivate women to undergo screening [7, 8]. Detecting BC cases in early stages has demonstrated to be detrimental for a low MPI. Countries such as Sweden, Norway, the United States and the UK present high percentiles regarding the early detection of BC and consequently, low mortality per case [9, 10]. Improvements on early detection for BC would improve Peru’s MPI since patients could receive timely treatment and improve their prognostic.

In order to further understand methods to improve Peru’s BC MPI, a study was conducted to evaluate women’s knowledge, attitude and barriers regarding BC and breast cancer screenings (BCS).

## Methods

### Study design

We conducted an observational, descriptive, transversal and nationwide pilot study to evaluate the level of knowledge, barriers and attitudes of Peruvian women regarding BCS.

### Study population and sample size

The study population included Peruvian women from 40 to 70 years old. The sample size of a minimum of 385 women was calculated with an expected proportion at 50% (worst-case scenario), 95% confidence and 5% margin of error using the Epidat 3.1 program. The sample was stratified to represent the access to the different types of insurance, Integral Health Insurance (SIS) 60%, Social Health Insurance (ESSALUD) 30% and private clinics 10%; as well as the distribution of the population in the evaluated departments.

In total, 488 participants were surveyed in nine departments that represent the north, middle and south of the country as well as the coastal, Andean and Amazonian regions, in national and private institutions. The departments were Lima, Cajamarca, Ica, Arequipa, Loreto, Ancash, Huancavelica, Junín and Madre Dios. The survey was conducted during September and October 2019.

### Selection criteria

We included women from 40 to 70 years old at the moment we applied the survey and, women with no current or past diagnosis of BC. On the contrary, we excluded women who go to a hospital with a BC patient, women with a condition that prevents them from reading the informed consent and, women who underwent unilateral or bilateral mastectomy for any reason.

### Survey design

We use the Rayyan QCRI platform (https://rayyan.qcri.org/welcome) to conduct a systematic review on PubMed, Scopus, Web of Science, Medline-Ovid and EMBASE; our strategy of search is on Supplementary Table 1.

We found 2,558 reports that assess the level of knowledge, barriers, attitudes or other aspects of BCS. We excluded case reports, editorials, narrative reviews and meta-analysis. Finally, 111 studies were selected.

We evaluated each of the selected studies and made a list of questions. Duplicate questions were removed and the remaining were sorted into categories: knowledge about BC, knowledge about BCS, barriers and attitudes. The list was evaluated by experts on the field and they decided which question was included or excluded. The final version of the survey was validated through Expert Judgement (two oncologists and a primary health care physician) and included 43 questions. Some questions were culturally adapted to be easily understood in Peru as for example, in the question about the suggested cost of the mammogram, the possible answers were in soles (Peruvian currency) instead of dollar.

In addition, we collected information about sociodemographic variables such as age, city of residence, residence at an urban or rural area, marital status (single, married/live-in partner and divorced/separated/widow), children (yes or no), level of education (primary or less, secondary and higher), employment status (unemployed, employed and retired) and type of insurance (SIS, ESSALUD or Armed forces, and private).

### Statistical analysis

Measures of central tendency and measures of dispersion were evaluated for quantitative data while frequencies and percentages were estimated for categorical data. The internal consistency of the questionnaire was evaluated according to the nature of each question; for questions about barriers and attitudes, the Cronbach’s alpha was used. Statistical analyses were performed using Statistical Package for the Social Sciences (SPSS^®^) software ver. 24.0 (IBM^®^, Armonk, NY, USA).

## Results

### General characteristics

In total, 488 women accepted to participate in the study; 30.7% were from Lima and 72.1% lived in an urban area. The mean of age was 53.3 (standard deviation: ± 9.1). Most of them were married or had a live-in partner (67.1%), and had children (93.6%). Regarding educational level, 37.0% had higher education. About half of participants were unemployed (51.2%) and 12.9% did not have any insurance (Table 1).

The internal consistency of the barriers and attitudes sections was good (Cronbach’s alpha: 0.793 and 0.779, respectively).

### Knowledge about BC and BCS

**Figure 1.**
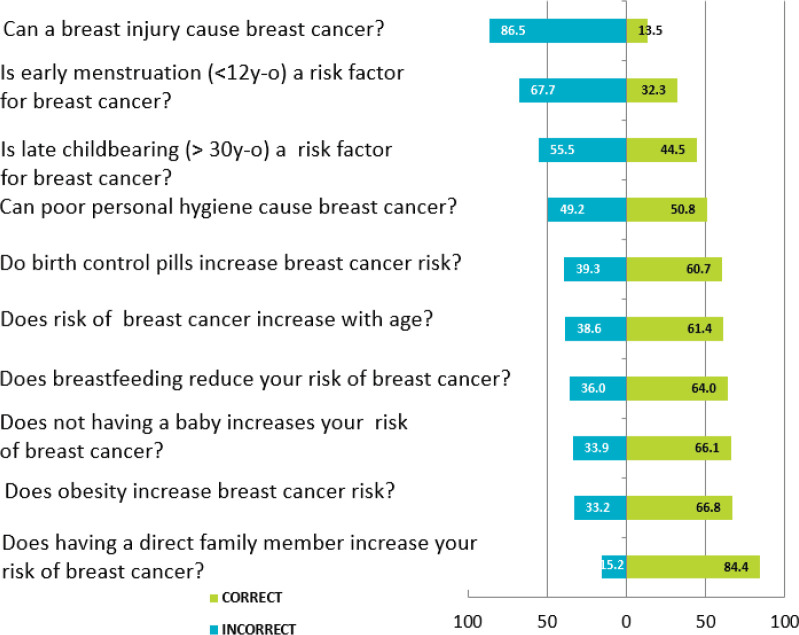
Knowledge about BC.

Most of the participants incorrectly believed that a breast injury can cause cancer (86.5%), and that start of menstruation before 12 years old was not a risk factor for BC (67.7%). In contrast, 84.4% were aware that having family history of BC increased their risk (Figure 1).

In total, 28.7% of women were unfamiliar with the term ‘mammogram’ and 47.1% never had a BCS (36.9% from urban and 73.5% from rural population). In women that underwent BCS, 67% knew it is for healthy women.

### Barriers

The most frequent reason to avoid mammogram was fear of the results (58.2% strongly agreed). For 39.6%, cost was an impediment to underwent screening and 44.5% reported that they never remember to make an appointment.

About half of patients (51.0%) preferred that a woman performed the exam and believed that mammogram was painful (48.8%). In addition, 37.5% considered that they had more important problems than getting a mammogram; 32.6%, that the staff are not very sensitive; 29.3%, that mammogram exposes them to unnecessary radiation; 20.3%, that they were too old to need examination and 17.5% that mammogram requires too much time.

Also, 30.2% of participants did not know what to do to get a mammogram, 29.7% were afraid because they did not know what to expect during the exam and 24.9% said that the felt ashamed of having a mammogram.

In total, 17% of women reported that their spouses did not like that a physician checked their breast.

### Attitudes

Regarding attitudes towards BCS (Figure 3), most of the participants strongly disagreed with the questions. However, 35.9% said that they preferred a breast ultrasound instead of mammogram, 35.3% think that there is no cure for cancer, 27.1% prefer not to know if they had BC and 20.1% believe that mammogram can cause cancer.

Remarkably, some women think that screening is not necessary since they do not have pain (25.7%), believe that they are at low risk (25.4%) and do not have any changes in their breasts (22.6%).

## Discussion

In this study, we explore the knowledge, barriers and attitudes of Peruvian women towards BCS. Although several efforts to enhance early detection of this neoplasm have been conducted, it is necessary to characterise our population in order to establish better strategies.

**Figure 2.**
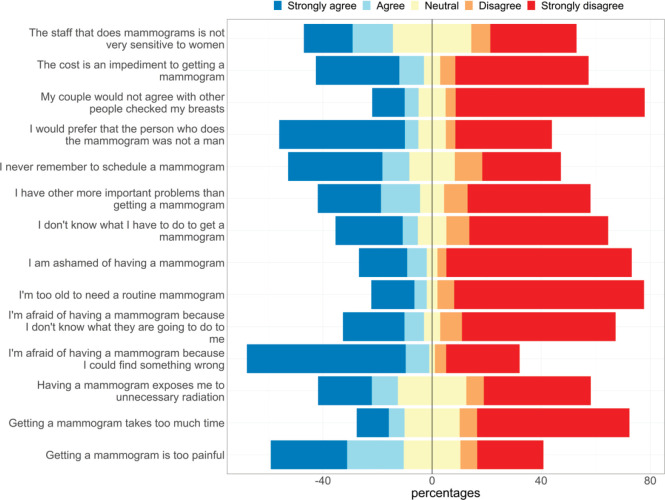
Barriers towards BCS.

**Figure 3.**
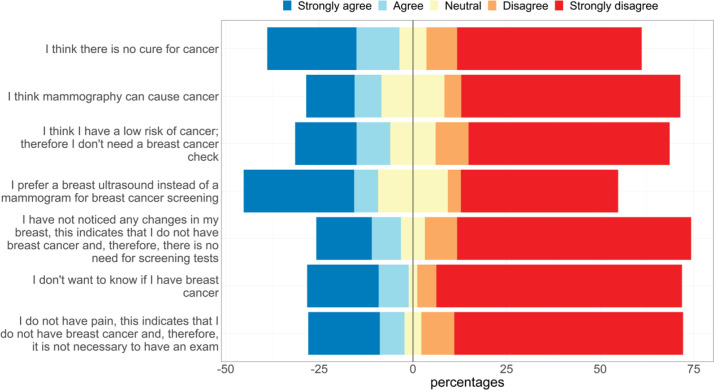
Perceptions towards BCS.

Our study revealed some misconceptions about risk factors for BC. Surprisingly, 86.5% of participants believed that a breast injury and poor hygiene (49.2%) can lead to BC. Also, 67.7% and 55.5% of women did not know that early menarche (before 12 years old) and bearing a first child after 30 years of age, respectively, increase the risk of BC. Educational campaigns are critical to educate our population and promote awareness of BC risk factors. Similar results were found in studies that included Hispanic and Jordanian women (Figure 2) [11, 12].

Regarding reasons why women prefer not to undergo BC screening, most of them were related to lack of knowledge about mammogram; such as considering that mammogram was painful or that it exposes them to excessive radiation. Misinformation about cancer screening and fear are critical factors that affect a considerable percentage of the target population. Therefore, it is crucial to improve patient education and clinicians and healthcare workers may play an important role in this movement [13].

An initiative similar to the Yo me cuido® Program could be carried out in Peru. This programme was initiated by the Moffitt Cancer Center to educate the Hispanic population living in the USA. The follow-up for mammography screenings showed that 52% of women had a screening mammogram within their first year after participating in the programme [14].

Another important factor to consider is the socioeconomic status; for 39.6%, cost was an impediment to undergo screening and, although it was not a question in the survey, participants living in the provinces manifest that the hospitals did not have the equipment and they were obligated to travel to other cities or visit private clinics if they wanted to have access to BCS. A study showed that Peruvian women with a higher level of education, higher wealth quintile and those from the capital city underwent screening mammogram in higher proportions; also, in a multivariate analysis, the highest wealth index quintile (prevalence ratio: 5.75; 95% confidence interval: 2.97–11.15) compared to the lowest quintile, was significantly associated with having a mammogram [15].

Unfortunately, Peruvian women still consider that if they do not have pain (25.7%) or any changes in their breast (22.6%), then they do not need to undergo screening, and 20.1% believe that a mammogram can cause cancer. Also 35.9% believed that there is no cure for cancer and 27.1% prefer not to know if they had cancer. This seems to be a frequent scenario in low-middle income countries. In Western Kenya, most of the interviewed women considered breast cancer a fatal disease and declared that they were afraid of having a mammogram because of a positive result [8]. In Ghana, the mean length of delay to attend diagnosis since presentations of sign or symptoms was 1.2 years, with a range of 2 weeks to 4 years. The main reasons were fear of mastectomy due to stigma, religion, myths and misconceptions about BC, no association between a painless breast lump with BC and financial reasons [16]. In Pakistan, 26% of the evaluated women reported that the reasons for delay in presentation were anxiety, fear and misconceptions about BC diagnosis and treatment and other social factors such as socioeconomic status and a negative impact on their relationship with their husband [17].

Altogether, these aspects may be the explanation as to why in Peru, most women are diagnosed when they manifest symptoms (93%) and only 6% by mammogram screening and 1% during screening clinical breast examination [18]. According to the Demographic and Family Health Survey (ENDES, in Spanish) 2017, only 17.1% of women between 40 and 59 years had a mammogram in the year prior to the survey; figures considerably lower compared to other countries where 50%–80% of the target population had had a mammogram [19–21].

Despite efforts from the Ministry of Health, a great proportion of cases are diagnosed in advanced stages. An analysis of patients diagnosed and treated at the Instituto Nacional de Enfermedades Neoplasicas (INEN) has shown that 35.9% of BC patients were stages III or IV at diagnosis in 2000–2002; that figure did not significantly change in the 2010–2012 period, where 36.7% were diagnosed with advanced BC [22, 23]. However, a recent study, conducted during the COVID-19 pandemic, has shown that 55.4% of cancer patients diagnosed and treated during 2019–2020 had an advanced stage [24]. In addition to the negative impact of COVID-19, the Peruvian healthcare system’s inequities have worsened BC early detection. Many women had to postpone their visit to oncological centres since they could not travel to the big cities where the facilities are located, or could not access telemedicine since they did not have Internet connection; therefore, diagnosis was postponed and mortality rates have increased [25, 26].

This study has some limitations. Because of its descriptive design, we cannot determine factors associated with the evaluated aspects in the study. However, our results are critical for a better design of strategies to encourage women to undergo BCS, especially considering that the COVID-19 pandemic has had a negative impact on cancer diagnosis.

## Conclusion

We found that it is feasible to conduct a large-scale study in Peru. The participants had misconceptions about BC and BCS. The results of this pilot study highlight an urgent need for extensive education and awareness about BCS in Peru.

## Conflicts of interest

The authors declare that they have no conflicts of interest.

## Funding statement

The study was funded by Oncosalud-AUNA.

## Author contributions

JMA: Methodology, formal analysis, investigation, data curation, writing – original draft, writing – review & editing, visualisation, project administration; ACG: Investigation, data curation, writing – original draft; WZDJ: Investigation, data curation, writing – original draft; JA: Investigation; IC: Investigation; LJS: Data curation; DB: Data curation; WF: Investigation; LGSH: Investigation; JBM: Methodology; ZM: Investigation; JRG: Investigation; HG: Conceptualisation; NKC: Investigation; LH: Investigation; WM: Investigation; CVG: Writing – review & editing; **C**C: Writing – review & editing; CP: Writing – review & editing; HG: Writing – review & editing; GM: Writing – review & editing; CF: Methodology; CV: Conceptualisation; JAP: Conceptualisation, methodology, formal analysis, supervision, funding acquisition.

## Figures and Tables

**Supplementary Table 1.** Search strategies in PubMed, Scopus and Web of Science (WOS).

	BÚSQUEDA EN LA BASE DE DATOS PUBMED
1	(“Breast Neoplasms” OR “Breast Neoplasm” OR “Neoplasm, Breast” OR “Breast Tumors” OR “Breast Tumor” OR “Tumor, Breast” OR “Tumors, Breast” OR “Neoplasms, Breast” OR “Breast Carcinoma” OR “Breast Carcinomas” OR “Carcinoma, Breast” OR “Carcinomas, Breast” OR “Mammary Neoplasms, Human” OR “Human Mammary Neoplasm” OR “Human Mammary Neoplasms” OR “Neoplasm, Human Mammary” OR “Neoplasms, Human Mammary” OR “Mammary Neoplasm, Human” OR “Breast Cancer” OR “Cancer, Breast” OR “Mammary Cancer” OR “Cancer, Mammary” OR “Cancers, Mammary” OR “Mammary Cancers” OR “Malignant Neoplasm of Breast” OR “Breast Malignant Neoplasm” OR “Breast Malignant Neoplasms” OR “Malignant Tumor of Breast” OR “Breast Malignant Tumor” OR “Breast Malignant Tumors” OR “Cancer of Breast” OR “Cancer of the Breast”)
2	(“Early Detection of Cancer” OR “Cancer Early Detection” OR “Cancer Screening” OR “Screening, Cancer” OR “Cancer Screening Tests” OR “Cancer Screening Test” OR “Screening Test” OR “Cancer Screening Tests” OR “Cancer Test, Cancer Screening Tests” OR “Cancer Screening” OR “Early Diagnosis of Cancer” OR “Cancer Early Diagnosis”)
3	#1 AND #2
4	(Questionnaires OR Surveys OR “Survey Methods” OR “Methods, Survey” OR “Survey Method” OR “Methodology, Survey” OR “Survey Methodology” OR “Community Surveys” OR “Community Survey” OR “Survey, Community” OR “Surveys, Community” OR “Repeated Rounds of Survey” OR Surveys OR Survey OR “Questionnaire Design” OR “Design, Questionnaire” OR “Designs, Questionnaire” OR “Questionnaire Designs” OR “Baseline Survey” OR “Baseline Surveys” OR “Survey, Baseline” OR “Surveys, Baseline” OR Respondents OR Respondent OR “Randomized Response Technique” OR “Randomized Response Techniques” OR “Response Technique, Randomized” OR “Response Techniques, Randomized” OR “Techniques, Randomized Response” OR Questionnaires OR Questionnaire OR Nonrespondents OR Nonrespondent)
5	(“Health Knowledge, Attitudes, Practice”)
6	#4 AND #5
7	#3 AND #6
	BÚSQUEDA EN LA BASE DE DATOS SCOPUS
1	ALL(“Breast Neoplasms” OR “Breast Neoplasm” OR “Breast Tumors” OR “Breast Tumor” OR “Breast Carcinoma” OR “Breast Carcinomas” OR “Human Mammary Neoplasm” OR “Human Mammary Neoplasms” OR “Breast Cancer” OR “Cancer, Breast” OR “Mammary Cancer” OR “Mammary Cancers” OR “Malignant Neoplasm of Breast” OR “Breast Malignant Neoplasm” OR “Breast Malignant Neoplasms” OR “Malignant Tumor of Breast” OR “Breast Malignant Tumor” OR “Breast Malignant Tumors” OR “Cancer of Breast” OR “Cancer of the Breast”)
2	ALL(“Early Detection of Cancer” OR “Cancer Early Detection” OR “Cancer Screening” OR “Cancer Screening Tests” OR “Cancer Screening Test” OR “Screening Test” OR “Cancer Screening Tests” OR “Cancer Screening” OR “Early Diagnosis of Cancer” OR “Cancer Early Diagnosis”)
3	#1 AND #2
4	ALL(Questionnaires OR Surveys OR “Survey Methods” OR “Survey Method” OR “Survey Methodology” OR “Community Surveys” OR “Community Survey” OR “Repeated Rounds of Survey” OR Surveys OR Survey OR “Questionnaire Design” OR “Questionnaire Designs” OR “Baseline Survey” OR “Baseline Surveys” OR Respondents OR Respondent OR “Randomized Response Technique” OR “Randomized Response Techniques” OR Questionnaires OR Questionnaire OR Nonrespondents OR Nonrespondent)
5	ALL(“Health Knowledge, Attitudes, Practice”)
6	#4 AND #5
7	#3 AND #6
	BÚSQUEDA EN LA BASE DE DATOS WOS
1	TS=(“Breast Neoplasms” OR “Breast Neoplasm” OR “Neoplasm, Breast” OR “Breast Tumors” OR “Breast Tumor” OR “Tumor, Breast” OR “Tumors, Breast” OR “Neoplasms, Breast” OR “Breast Carcinoma” OR “Breast Carcinomas” OR “Carcinoma, Breast” OR “Carcinomas, Breast” OR “Mammary Neoplasms, Human” OR “Human Mammary Neoplasm” OR “Human Mammary Neoplasms” OR “Neoplasm, Human Mammary” OR “Neoplasms, Human Mammary” OR “Mammary Neoplasm, Human” OR “Breast Cancer” OR “Cancer, Breast” OR “Mammary Cancer” OR “Cancer, Mammary” OR “Cancers, Mammary” OR “Mammary Cancers” OR “Malignant Neoplasm of Breast” OR “Breast Malignant Neoplasm” OR “Breast Malignant Neoplasms” OR “Malignant Tumor of Breast” OR “Breast Malignant Tumor” OR “Breast Malignant Tumors” OR “Cancer of Breast” OR “Cancer of the Breast”)
2	TS=(“Early Detection of Cancer” OR “Cancer Early Detection” OR “Cancer Screening” OR “Screening, Cancer” OR “Cancer Screening Tests” OR “Cancer Screening Test” OR “Screening Test” OR “Cancer Screening Tests” OR “Cancer Test, Cancer Screening Tests” OR “Cancer Screening” OR “Early Diagnosis of Cancer” OR “Cancer Early Diagnosis”)
3	#1 AND #2
4	TS=(“Early Detection of Cancer” OR “Cancer Early Detection” OR “Cancer Screening” OR “Screening, Cancer” OR “Cancer Screening Tests” OR “Cancer Screening Test” OR “Screening Test” OR “Cancer Screening Tests” OR “Cancer Test, Cancer Screening Tests” OR “Cancer Screening” OR “Early Diagnosis of Cancer” OR “Cancer Early Diagnosis”)
5	TS=(“Health Knowledge, Attitudes, Practice”)
6	#4 AND #5
7	#3 AND #6

**Table 1.** Demographic characteristics of surveyed women (*n* = 488).

Characteristics	*n* (%)
Age mean (SD)	
53.3 (±9.1)	
Department Lima	150 (30.7)
Cajamarca	93 (19.1)
Ica	59 (12.1)
Arequipa	56 (11.5)
Loreto	48 (9.8)
Ancash	38 (7.8)
Huancavelica	14 (2.9)
Junín	15 (3.1)
Madre de Dios	15 (3.1)
Area of residence Urban	352 (72.1)
Rural	136 (27.9)
Marital status Married/live-in partner	326 (67.1)
Single	83 (17.1)
Separated/widower	77 (15.8)
No response	2
Children No	27 (5.6)
Yes	457 (94.4)
Without response	4
Education Primary or less	145 (29.8)
Secondary	162 (33.2)
Higher than secondary	180 (37.0)
Without response	1
Employment status Unemployed	250 (51.5)
Employee	217 (44.7)
Retired	18 (3.7)
Without response	3
Insurance Yes No	425 (87.1) 63 (12.9)
Type of insurance SIS	237 (55.8)
EsSalud/Armed Forces	145 (34.1)
Private insurance	43 (10.1)
SD, Standard deviation	
